# A case of eosinophilic granulomatosis with polyangiitis complicated with a similar condition to IgG4 related lung disease

**DOI:** 10.1186/s12890-019-0917-4

**Published:** 2019-08-19

**Authors:** Li Zhou, Fen Cao, Songqing Fan, Ping Chen, Shuizi Ding, Guiqian Liu, Ruoyun Ouyang

**Affiliations:** 1 Department of Pulmonary and Critical Care Medicine, The Second Xiangya Hospital, Central South University, 139 Renmin Middle Road, Changsha, 410011 Hunan China; 2 Research Unit of Respiratory Disease, Central South University, 139 Renmin Middle Road, Changsha, 410011 Hunan China; 3 Diagnosis and Treatment Center of Respiratory Disease, Central South University, 139 Renmin Middle Road, Changsha, 410011 Hunan China; 4 Department of Pathology, The Second Xiangya Hospital, Central South University, Changsha, China

**Keywords:** EGPA, IgG4-RD, Elevated serum IgG4, IgG4 positive plasma cell, Pathology of lung tissue

## Abstract

**Background:**

Atypical manifestations, such as elevated serum immunoglobulin-G4 (IgG4) and extra-pulmonary IgG4 positive plasmacyte infiltration, have been described in patients with eosinophilic granulomatosis with polyangiitis (EGPA), such complicated situation might not be readily differentiated from IgG4-related disease.

**Case presentation:**

Here, we report an interesting and rare case of EGPA in a 41 year-old male with negative anti-neutrophil cytoplasmic antibodies (ANCAs), which showed abundant pulmonary IgG4-positive plasma cells infiltration and markedly elevated serum IgG4 levels without extra-pulmonary lesions of IgG4-related disease. The clinical characteristics hesitated us whether the diagnosis as EGPA overlapping with IgG4-related lung disease should be concluded because of the absence of storiform fibrosis and obliterative phlebitis with lymphoplasmacytic infiltration. The patient’s systemic symptoms, pulmonary lesions, blood eosinophils count / percentage, and serum IgG4 levels were significantly improved with immunosuppressive therapy.

**Conclusions:**

We consider that the overlapping pathogenesis exists in the disease course of EGPA and IgG4-related disease, which may lead to interaction during the course of the diseases. And the utilization of diagnostic criteria for IgG4-related lung disease with the careful differentiation is needed in such cases.

## Background

Eosinophilic granulomatosis with polyangiitis(EGPA), previously called Churg-Straus syndrome, is a rare systematic disorder histopathologically characterized with eosinophilic infiltration, extravascular granulomas and necrotizing vasculitis predominantly affecting small to medium-sized vessels [1]. Recent research described the epidemiologic and demographic features of EGPA which showed a prevalence rates of two to 22.3 per million and the annual incidence rates of 0.5–3.7 per million and incidence peak occurred at the age of 30 to 40 or 55 to 64 year-old [2]. The clinical manifestations of EGPA are involved with severe asthma, allergic rhinitis, blood and tissue eosinophilia with cardiac, gastrointestinal, skin, renal involvement and peripheral neuropathy. And EGPA is classically considered as a Th2-mediated disease and can be subclassified as antineutrophil cytoplasmic antibodies (ANCAs) positive, which are only found in 30–40% patients with EGPA, and ANCA-negative EGPA [3, 4]. However, the accurate diagnosis of EGPA is often difficult, because of the similar or overlapping clinical manifestations to chronic eosinophilic pneumonia, hypereosinophilic syndrome, other primary systemic vasculitis, and hyper-immunoglobulin G4 syndrome [5]. IgG4-related disease (IgG4-RD) is a novel systemic immune-mediated fibro-inflammatory condition involving multiple organs, and characterized by markedly increased serum IgG4 level, lymphoplasmacytic infiltration with abundant IgG4-positive plasmacytes, storiform fibrosis and obliterative phlebitis [6]. However, clinical data found increased level of serum IgG4 and/or elevated serum IgG4/IgG ratio in patients with active EGPA. And increases in IgG4 positive plasma cells were also found in the tissue biopsies from patients with EGPA [5]. Similarity between these two diseases often causes the diagnostic dilemma to differentiate them. Herein, we report a case of EGPA patient with a pathological condition similar to IgG4-related lung disease and discuss the similarity and the key difference between these two conditions. The report aims to improve the awareness of these two rare clinical diseases and prevent the diagnostic dilemma in clinical practice.

## Case presentation

### Clinical history

A 41-year-old Chinese male was admitted to our hospital because of a 3-year history of recurrent productive cough and new onset of recurrent hemoptysis and fever for 6 months. Three years ago, the patient had been admitted to the local hospital because of cough and expectoration, and the chest computed tomography (CT) scan revealed bilateral lower lung infection. Not much improvement of respiratory symptoms had been observed, although treatments of anti-infection and anti-tuberculosis had been administrated in turn. Besides, the patient suffered a new onset of intermittent symmetric pain of limb joints, swelling of the upper eyelid, and erythematous maculopapular rash on the dorsal surfaces of the metacarpophalangeal joint, bilateral elbow joint, and proximal interphalangeal joint. Both lower limbs developed a livedo reticularis appearance after standing for approximate 5 min. Six months before admission, the patient experienced hemoptysis and developed a fever with maximum temperature of 38.5 °C. The frequency of redness and swelling of upper eyelids increased with and left and right eyelids alternated once a week. The new soybean-sized subcutaneous nodules with tenderness were found on the bilateral finger pulp. High potency anti-infection, anti-tuberculosis and anti-fungus treatment were given but demonstrated ineffectiveness with accelerated cough and expectoration. The patient had a 3-year history of sinusitis and family history of asthma. And he had no previous history of smoking and drinking.

### Physical and laboratory examinations

**Fig. 1:**
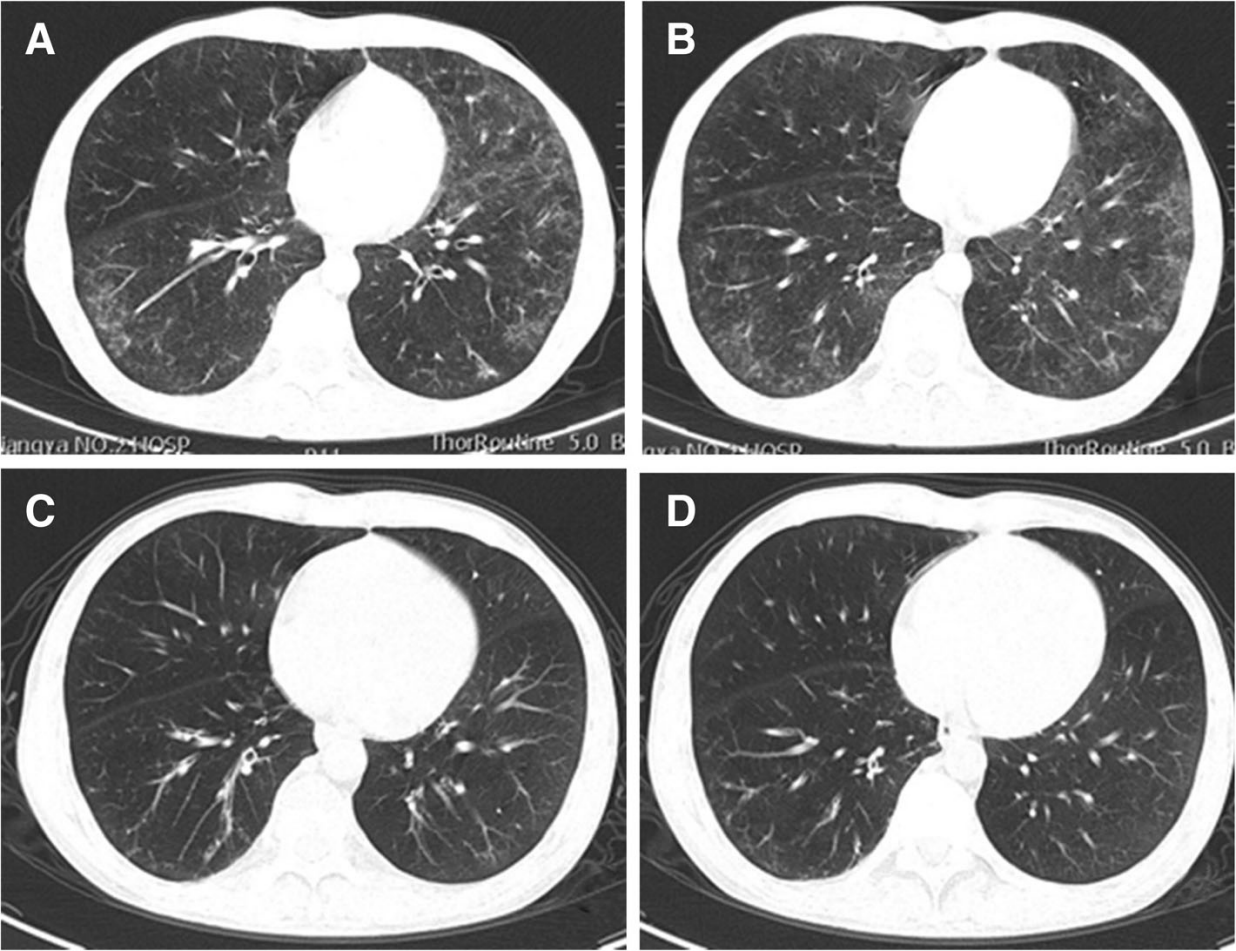
**a**-**b** Chest CT scan on admission showed increased bilateral pulmonary texturesand local mosaic perfusion. And diffuse ground-glass opacities and patches with increased density and blurry edges presented in both lungs. **c**-**d** One month after immunosuppressive therapy, chest CT scan showed absorption and marked improvement of pulmonary lesions

**Fig. 2:**
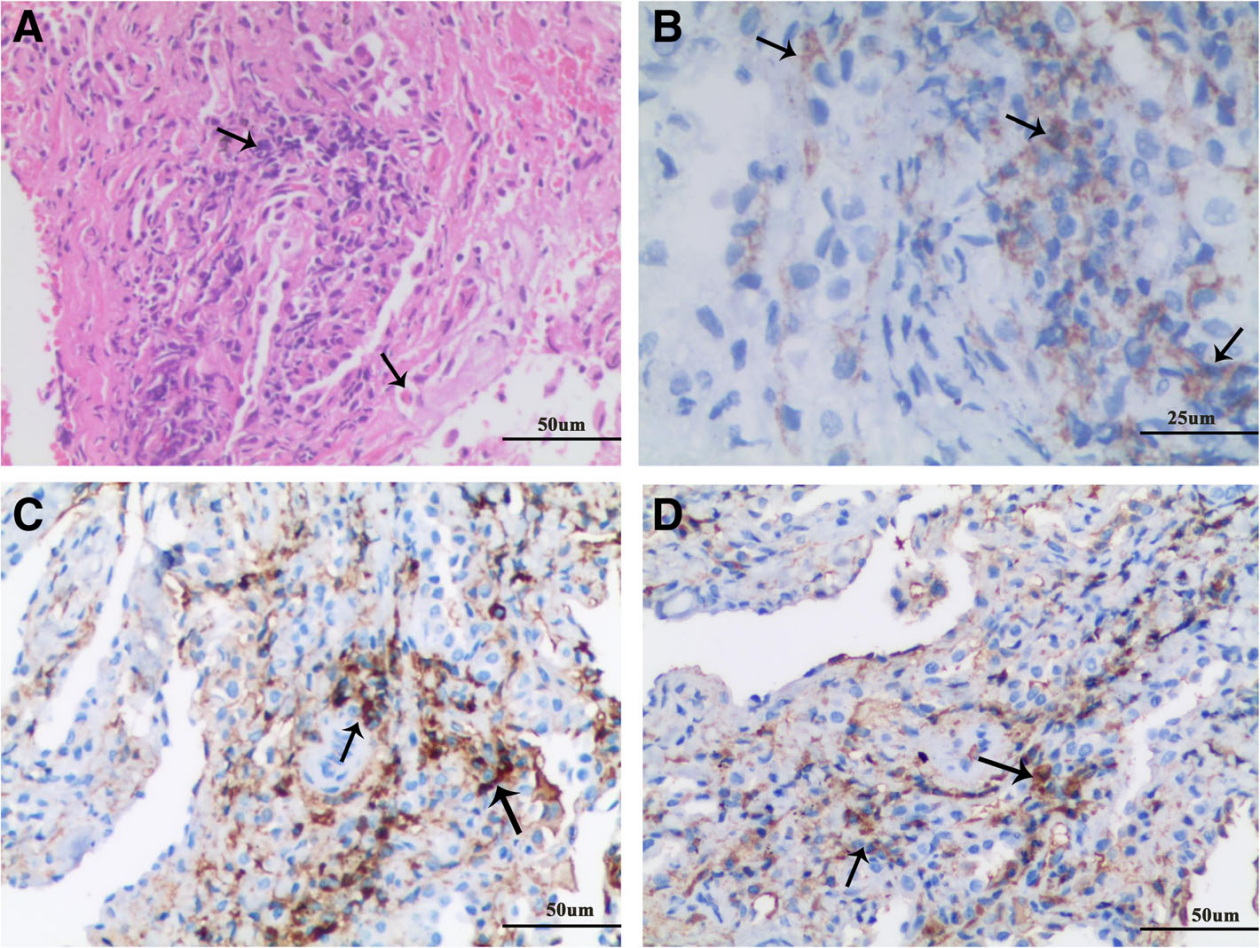
Lung biopsy: **a** Arrows indicate infiltration of lymphocytes, histiocytes, and eosinophils in the lung tissue (HE staining; magnification 200×). **b** Immunohistochemistry study for CD138 showed positive CD138 cells-plasma cells (arrow; DAB staining; magnification 400×). **c** Immunohistochemical examination with IgG-specific antibody for specimens from lung biopsy, showing positive infiltration of IgG plasma cells (arrow; magnification 400×); **d** Immunohistochemical examination with IgG4-specific antibody for specimens from lung biopsy, indicating enriched IgG4+ plasma cell infiltration. (arrow; magnification 200×). HE, hematoxylin-eosin; as indicated by the arrows

**Fig. 3:**
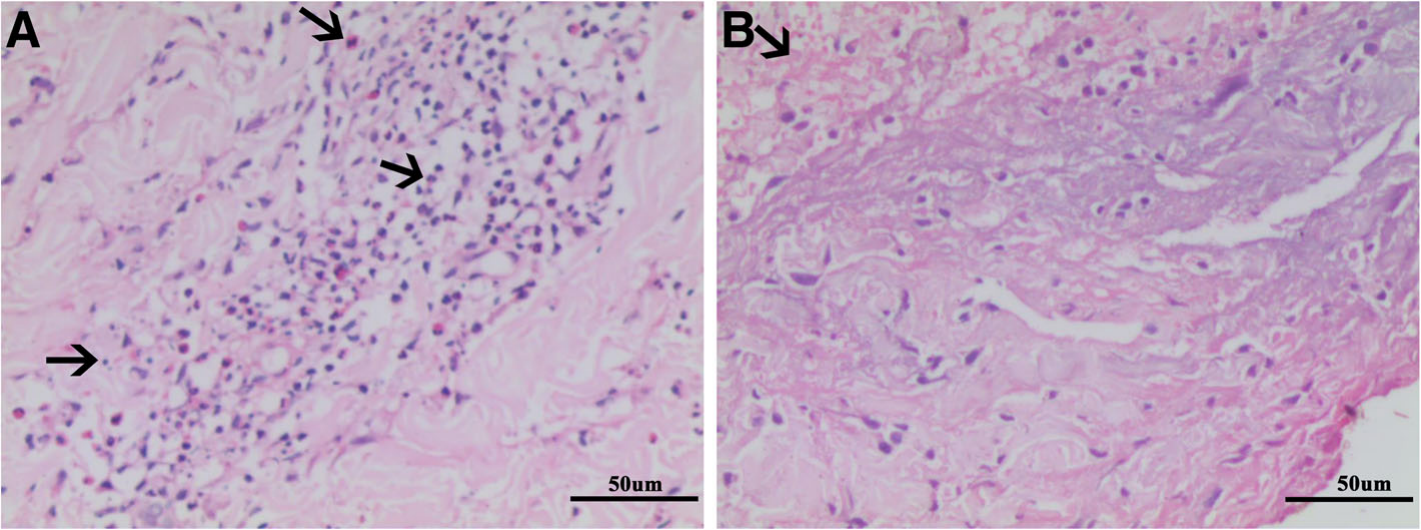
Skin biopsy: **a** Infiltration of eosinophils, neutrophils, and lymphocytes presented in the vasculature and interstitial spaces of the dermal layer (arrows; HE staining; magnification 200×); **b** The arrow showed focal fibrinoid necrosis in the reticular dermis (HE staining; magnification 200×)

Physical examination showed redness and swelling of right eyelid, and gastrocnemius tenderness. And fine moist rales and wheezes were heard in both lower lung fields. An erythematous maculopapular rash on the dorsal surfaces of the metacarpophalangeal joint of the right hand and dark red pigmentation on the right elbow were found. Laboratory studies revealed lightly elevated leukocyte count (10.73 × 10^^9^/L), and markedly increased eosinophil count and percentage (1.26 × 10^^9^/L, 11.7%), erythrocyte sedimentation rate (99 mm/h) and C-reactive protein (167 mg/L). Further laboratory studies showed increased levels of serum IgG (30.8 g/L) and highly elevated levels of serum IgG4 (24.5 g/L). Tests for IgE levels, complement system, Anti-nuclear antibody, anti-extractable nuclear antigen antibody, dermatomyositis-associated antibodies, myeloperoxidase-ANCA, proteinase-3-ANCA, anti-cardiolipin antibody, anti-cyclic citrullinated peptide antibody and rheumatoid factor were all normal. The ultrasounds of parotid gland, submandibular gland, thyroid, heart were all normal. Pulmonary function tests showed severe mixed pulmonary ventilation dysfunction, moderate pulmonary diffusion function,negative bronchodilation test. And the diurnal variation of peak expiratory flow (PEF) was more than 10% and the value of fractional exhaled nitric oxide was also high (26 ppb). A chest CT scan revealed diffuse ground-glass opacities in both lungs and patches with increased density and blurred edges (Fig. 1a-b). A CT scan of the sinuses showed ethmoid sinusitis on the left side and maxillary sinusitis bilaterally. Bronchoscopy did not find any abnormalities and bronchoalveolar lavage fluid showed normal cellular counts and eosinophil count. Bone marrow puncture showed active proliferation of bone marrow cells, in which the proportion of osinophils was 12%, and no abnormal pathological cells were observed. The genetic analyses showed normal FIP1L1/PDGFRα and ETV6-PDGFRβ fusion genes. Pathological examination of lung biopsy showed fibrosis, infiltration of lymphocytes, plasma cells and histocytes, and the presence of eosinophils in small vessels of bronchiolar walls. Immunohistochemistry revealed CD38+, CD138+, IgG+, and IgG4+ cells in which the ratio of IgG4+ to IgG+ plasma cells was greater than 40% and there were more than 10 IgG4+ plasma cells per high-power field (Fig. 2). The skin biopsy showed enriched infiltration in the vasculature and interstitial spaces of the whole dermis by eosinophils, neutrophils, and lymphoid tissue, along with focal fibrinoid necrosis in the reticular dermis (Fig. 3).

### Diagnostic basis

There are no commonly accepted diagnostic criteria for EGPA. And the classification criteria for EGPA defined by American College of Rheumatology (ACR) was widely used by clinician [7]. It is composed of six criteria for EGPA: (1) asthma, (2) eosinophilia>10%, (3) neuropathy(mono- or poly-neuropathy), (4) non-fixed pulmonary infiltrates, (5)paranasal sinus abnormality, (6) extravascular eosinophil infiltration on biopsy finding. When four or more of the above six criteria are present, vasculitis can be classified as EGPA with a sensitivity of 85% and specificity of 99.7%. Our patient had typical symptoms (cough, shortness of breath) and physical sign (wheezes in lower lungs) of asthma for more than 3 years. And the average daily diurnal PEF variability was more than 10%. Besides, the patient had the family history of asthma plus with high value of fractional exhaled nitric oxide. Therefore, the diagnosis of asthma was established. The possible cause for the negative bronchodilation test is that the patient was taking inhaled budesonide-formoterol (inhaled corticosteroids-long-acting beta_2_-agonist drug, ICS-LABA) medication to relieve shortness of breath before admission and during hospitalization. Furthermore, the percentage of blood eosinophil was 11.7%, which was more than 10%. The patient also had a 3-year history of sinusitis, which was also confirmed by CT scan of sinuses. The chest CT scan showed non-fixed pulmonary infiltration, with multiple patches in both lungs and ground-glass opacities. Moreover, the skin biopsy showed enriched eosinophil infiltration in the vessels and interstitial spaces of the dermis. Therefore, the patient was diagnosed as EGPA with following diagnostic basis: asthma, peripheral blood eosinophilia (>10%), paranasal sinusitis, transient pulmonary infiltration and eosinophil infiltration of the skin.

### Treatment

We discontinued all of the patient’s medications which had been prescribed before admission. Immunosuppressive treatment with intravenous methylprednisolone (80 mg × 3d, 40 mg × 3d)was started and followed by oral methylprednisolone (40 mg/d). Seven days later, respiratory symptoms, like cough and expectoration, were improved. Hemoptysis, fever, redness and swelling of eyelid and polyarthralgia were all completely disappeared. The follow-up laboratory tests showed reduced eosinophils count and percentage (0.07 × 10^^9^/L, 0.40%), and serum IgG4 level (16.6 g/L). After discharge, treatment of oral methylprednisolone with 35 mg/d and intravenous cyclophosphamide (0.4 g per 2 week) were both administrated. One month later, cough, expectoration, maculopapular rash, subcutaneous nodules, reticulated blemishes were all completely disappeared. Gastrocnemius tenderness was also reduced. The follow-up laboratory tests showed further decreased eosinophils count and percentage (0.01 × 10^^9^/L, 0.10%) and serum IgG4 level (7.41 g/L). The follow-up thoracic CT scan showed that bilateral lung lesions were also significantly improved (Fig. 1c-d). Summary of the patient’s symptoms and corresponding treatment was described in the Table 1.

**Table 1:** Summary of the patient’s symptoms, applied treatment and improvement rating

Timeline	Symptoms	Treatment	Improvement rating
2013	Productive cough	Repeated anti-infection therapy	Re-occurrence & exacerbation
March, 2016	Exacerbation in cough, dyspnea, hemoptysis, recurrent fever, redness, swelling of eyelid and polyarthralgia	All kinds of Antibiotics therapy (Piperacillin/tazobartan, cefmenoxime, meropenem, levofloxacin etc.) Anti-tuberculosis therapy Antifungal therapy	No improvement & exacerbation
Sep 14–18, 2016	Exacerbation in cough, dyspnea, hemoptysis, recurrent fever, redness, swelling of eyelid and polyarthralgia	Piperacillin/tazobartan, Amikacin, roxithromycin	No improvement
Sep 19–30, 2016	Productive cough, recurrent fever, hemoptysis, dyspnea, swelling of eyelid and polyarthralgia	cefoperazone/sulbactam, levofloxacin, azithromycin	No improvement
Oct 1–7, 2016	Increased productive cough, recurrent fever, swelling of eyelid and polyarthralgia	Levofloxacin, roxithromycin	No improvement
Oct 8–12,2016	Productive cough, subxiphoid pain, sore throat, dyspnea, recurrent fever, swelling of eyelid and polyarthralgia	Ceftazidime, amikacin, roxithromycin	No improvement
Oct 12–18,2016	Productive cough, subxiphoid pain, sore throat, dyspnea, recurrent fever, swelling of eyelid and polyarthralgia	intravenous methylprednisolone	Significant improvement or disappearance in symptoms and laboratory examinations
From Oct 19, 2016	Some cough and expectoration	Oral methylprednisolone & intravenous cyclophosphamide	Further obvious improvement and disappearance in symptoms and laboratory examinations

## Discussion and conclusion

The present report shows a rare case of EGPA complicated with marked elevation of serum IgG4 concentration and abundant IgG4+ plasmacytes infiltration in the lung tissue, which lead to the a diagnostic difficulty regarding whether IgG4-related lung disease should be concluded to this patient. The updated comprehensive diagnostic criteria for IgG4-RD includes: (1)clinical manifestation with typical diffuse/localized swelling or masses in single/multiple organs; (2)elevated serum IgG4 concentrations(>1.35 g/L); (3) characteristic histopathological examination showing significant lymphocytes and plasmacytes infiltrate, fibrosis and enriched infiltration of IgG4+ plasmacytes with ratio of IgG4+ to IgG+ cells >40% and >10 IgG4+ plasma cells per high-power field [8]. Definite IgG4-RD was established by suggestive organ involvement, combination with highly increased serum IgG4 levels and characteristic histological manifestations. However, storiform fibrosis and obliterative phlebitis with lymphoplasmacytic infiltration, two specific histopathological features in the patients with IgG4-RD, and involvement of other organs (lacrimal or salivary gland, lymph nodes, pancreas or kidney etc.) were absent in our case, which hesitated us whether it is reasonable to give a diagnosis of IgG4-RD simultaneously. No organ-specific diagnostic criteria has been established for IgG4-related lung disease. Markedly elevated serum IgG4 level and enriched infiltration of IgG4+ plasma cells in this patient may implicate the diagnosis for IgG4-RD, but we could not confirm whether eyelids swelling and lung lesions (diffuse ground glass, patchy density) were resulted from IgG4-RD.

In recent years, elevation of serum IgG4 level and/or IgG4+ plasma cells infiltrate were found in multiple lung diseases, such as pulmonary malignancy, granulomatosis with polyangiitis, EGPA, interstitial pneumonias, multicentric castleman’s disease [6]. A high ratio of IgG4 to IgG positive plasma cell (>40%) and >50 IgG4+ plasma cells in a high-power filed without extra-thoracic manifestation of IgG4-RD have been reported in 5 patients with surgical lung biopsy-proved idiopathic interstitial pneumonia in a retrospective study [9]. EGPA may be also accompanied by elevated serum IgG4 levels. Several studies have showed elevated serum IgG4 level [10, 11] and/or infiltration of IgG4-positive plasma cell in the tissue biopsy, two crucial features in patients with IgG4-RD, among EGPA patients [12, 13]. Nobuhiro et al. described a EGPA case with a similar clinical condition of IgG4-related kidney disease, in which the patient was involved with a high serum IgG4 level, salivary gland swelling, tubulointerstitial nephropathy and infiltrate of IgG4 positive plasmacytes in the renal tissue, although the ratio of IgG4+ plasma cells to all IgG+ plasma cells is about 10% (less than 40%). Suguru et al. presented a case showing numerous infiltration of IgG4-positive plasma cells (about 50%) in the right upper eyelid and high serum IgG4 concentration (119 mg/dl) during the disease course of EGPA [13]. Besides, the ratio of serum IgG4 to IgG and IgG4 levels were elevated in the disease course. Furthermore, a study found that there was no significant difference in IgG subclasses between IgG4-RD and EGPA [11]. A European retrospective multicenter observational study in patients with diagnosis of ANCA-associated vasculitides and IgG4-RD concomitantly showed that lymphoplasmacytic infiltration with IgG4/IgG ratio>40% and/or> 10 IgG+ plasma cell per high-power field was noted in the kidney, orbital mass or aorta biopsy [14]. On the other hand, studies showed that up to 50% of patients with IgG4-RD have a clinical history of allergic condition, such as asthma, sinusitis [15]. Marked eosinophilia and highly elevated IgE levels, which are features of EGPA, have also been commonly reported in patients with IgG4-RD. [16, 17] All above studies demonstrated a diagnostic dilemma and overlapped immunopathogenetic mechanisms existed in the disease course of these two diseases. Or whether IgG4-RD can be developed from EGPA?

Up-regulated responses of T helper (Th) 2 cell and T regulatory (Treg) cells and their cytokines (interleukin (IL)-4, IL-5, IL-13, IL-10 and transforming growth factor beta 1) play a key role in the pathogenesis of IgG4-RD. [15] EGPA is classically considered as a Th2 cellular-mediated immune-inflammatory disease, in which high levels of Th2-associated cytokines (IL-4, IL-5 and IL-13) are secreted and precipitates a B-cell response resulting in the production of IgE, IgG4 and ANCA and simultaneously increases expression and secretion of eotaxin-3 guiding eosinophil to the endothelium and tissues resulting in the eosinophilia and damage of organs [4]. The cytokines IL-4, IL-5, IL-13 have been demonstrated to be involved in triggering the production of IgE or generation of IgE-producing plasma cells and promoting the production of IgG4 [18]. Besides, IgG4, a Th2-dependent IgG subclass, and IgE both can be induced by allergens [19]. More importantly, IgE can be switched into IgG4 under the effect of IL-4 and IL-13, which indicates elevated levels of IgG4 in the Th2-immune responses may be associated with the increases of IgE. Above evidences seem to well explain, to some extent, why EGPA and IgG4-RD share common clinical manifestations, like asthma and allergy, markedly elevated IgG4 levels, and abundant infiltration of IgG4 positive plasma cells. And we consider whether our patient was just during the early stage of IgG4-RD. Or maybe EGPA interact with IgG4-RD and the disease course of EGPA can promote the onset of IgG4-RD due to the overlap pathogenesis.

In summary, giving a diagnosis of IgG4-RD may be challenging in such cases and requires a comprehensive consideration with clinical symptoms, laboratory examinations and histopathologic studies. We gave the patient the diagnosis of EPGA complicated with probable IgG4-RD eventually. Fortunately, recommended first-line therapy for EPGA and IgG4-RD are both immunosuppressive therapy with glucocorticoids [6, 20]. In our case, the patient received intravenous methylprednisolone and adjuvant treatment with intravenous pulses of cyclophosphamide because of involvement of multiple organs. The patient’s pulmonary and systemic symptoms, pulmonary lesions, laboratory tests for blood eosinophil count and serum IgG4 levels were all significantly improved with above treatment.

In conclusion, it is the first report showing markedly elevated serum IgG4 levels and abundant infiltration of IgG4 positive plasma cells in the lung tissue simultaneously in patient with EGPA, which strongly suggest us that EGPA and IgG4-RD may share an overlapped pathogenesis in local and systemic IgG4 response. We consider that EGPA and IgG4-RD may intersect in the course of disease and, in some extent, the development of one disease can influence the onset of another disease. Further studies should be required to investigate the potential mechanism.

## Data Availability

All the data discussed in the manuscript are included within this published article.

## Abbreviations

ANCA: Antineutrophil cytoplasmic antibodies
CT: Computed tomography
EGPA: Eosinophilic granulomatosis with polyangiitis
IgG4: Immunoglobulin-G4
IgG4-RD: IgG4-related disease
IL: Interleukin
